# Supplementary material for: The Evolution and Characterization of the RNA Interference Pathways in Lophotrochozoa
Source: Genome Biol Evol. 2024 May 7;16(5):evae098. doi: 10.1093/gbe/evae098 (PMC11114477; doi:10.1093/gbe/evae098)
Supplement: evae098_Supplementary_Data [file evae098_supplementary_data.zip › Supplementary_materials.zip!/selected_miRNAs/NoGe_selected_miRNAs/mirdeep_pdf_files/NMRB01001123.1_Notospermus_geniculatus_strain_Ushimado_scaffold1123,_whole_genome_shotgun_sequence_25191.pdf]

Provisional ID	:	NMRB01001123.1_Notospermus_geniculatus_strain_Ushimado_scaffold1123,_whole_genome_shotgun_sequence_25191
Score total	:	1127.3
Score for star read(s)	:	3.9
Score for read counts	:	1123.8
Score for mfe	:	0.2
Score for randfold	:	0
Score for cons. seed	:	-0.6
Total read count	:	2216
Mature read count	:	1967
Loop read count	:	0
Star read count	:	249

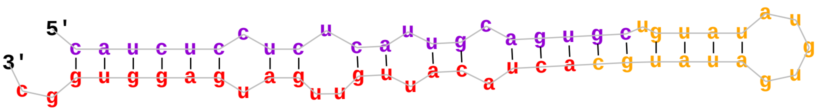

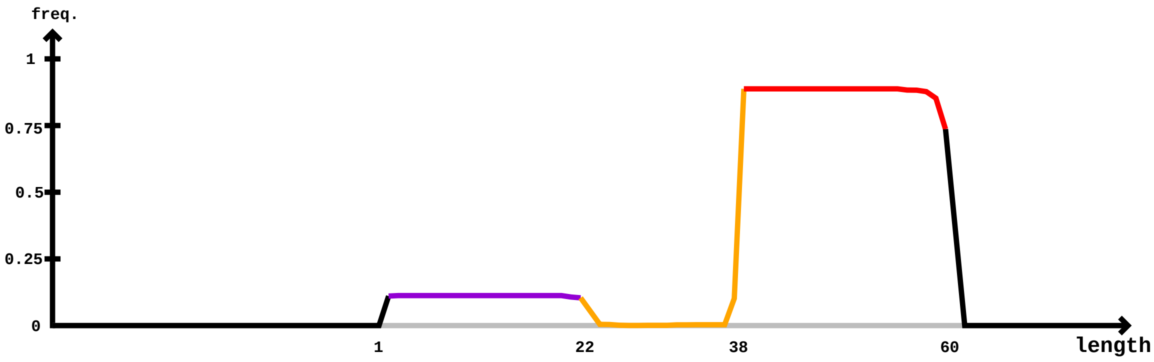

Star Mature

```
5'- agacaguggaaaugaagcagcagcuagcuuucucaucuccucucauugcagugcuguauaugugauaugcacuacauuguugaugagguggcaauguuggcuuuuaucau  -3'   obs
    agacaguggaaaugaagcagcagcuagcuuucucaucuccucucauugcagugcuguauaugugauaugcacuacauuguugaugagguggcaauguuggcuuuuaucau         exp
    ...........((((...((.(((((((.((.(((((((.((.((.((.(((((.((((.....))))))))).)).)).))..)).))))))).)).))))))).)).))))    reads    mm    sample
    .................................caucuccucucauugcagugc........................................................    1        0     Mm3
    ......................................................................acuaAauuguugaugagguggc..................    1        1     Mm3
    ......................................................................acuacauuguugaugagguggc..................    1        0     Mm3

    .................................caucuccucucauugcagug.........................................................    4        0     Mm1
    .................................Uaucuccucucauugcagugc........................................................    1        1     Mm1
    .................................caucuccucucauugcagugc........................................................    20       0     Mm1
    ......................................................................acuacauuguugaugagguggc..................    14       0     Mm1

    .................................caucuccucucauugcagugc........................................................    18       0     Mm2
    .................................caucuccucucauugcagugcu.......................................................    7        0     Mm2
    .................................caucuccucucauugcagugcG.......................................................    2        1     Mm2
    .................................Uaucuccucucauugcagugcu.......................................................    2        1     Mm2
    ...............................................................gauaugcacuacauuguugaugaggu.....................    2        0     Mm2
    .....................................................................cacuacauuguugaugagguggc..................    6        0     Mm2
    ......................................................................acuacauuguugaugaggA.....................    1        1     Mm2
    ......................................................................acuacauuguugaugaggug....................    2        0     Mm2
    ......................................................................acuacauuguugaugagAugg...................    1        1     Mm2
    ......................................................................acuacauuguugaugaggugg...................    41       0     Mm2
    ......................................................................acuacauAguugaugaggugg...................    1        1     Mm2
    ......................................................................aAuacauuguugaugagguggc..................    1        1     Mm2
    ......................................................................acuacauuguugaugagguggU..................    8        1     Mm2
    ......................................................................acuacauuguugaugagguggc..................    164      0     Mm2
    ......................................................................acuacauuguugaugagAuggc..................    1        1     Mm2
    ......................................................................acuacauuguugaugagguggG..................    1        1     Mm2
    ......................................................................acuacauuguugaugagUuggc..................    1        1     Mm2
    ......................................................................Ucuacauuguugaugagguggc..................    1        1     Mm2
    ......................................................................acuacauuguCgaugagguggc..................    1        1     Mm2
    ......................................................................acuacauuguugaugagguggcU.................    8        1     Mm2
    ......................................................................acuacauuguugaugagguggcC.................    1        1     Mm2
    ......................................................................acuacauuguugaugagguggca.................    1        0     Mm2

    .................................cGucuccucucauugcagu..........................................................    1        1     Fm3
```

Star **Mature**

```
agacaguggaaaugaagcagcagcuagcuuucucaucuccucucauugcagugcuguauaugugauaugcacuacauuguugaugagguggcaauguuggcuuuuaucau
.................................caucuccucucauugcagu..........................................................   10    0   Fm3
.................................caucuccucucauugcaUug.........................................................   1     1   Fm3
.................................caucuccucucauugcagug.........................................................   2     0   Fm3
.................................caucuccucucauugcagugc........................................................   73    0   Fm3
.................................Uaucuccucucauugcagugc........................................................   2     1   Fm3
.................................caucuccucuUauugcagugc........................................................   2     1   Fm3
.................................cCucuccucucauugcagugc........................................................   2     1   Fm3
.................................Aaucuccucucauugcagugc........................................................   1     1   Fm3
.................................caucuccucucauugcaAugc........................................................   1     1   Fm3
.................................caucuccucucauugcaUugc........................................................   2     1   Fm3
.................................caucuccucucauugcagugU........................................................   3     1   Fm3
.................................caucuccucucauugcagugcu.......................................................   48    0   Fm3
.................................Uaucuccucucauugcagugcu.......................................................   1     1   Fm3
.................................caAcuccucucauugcagugcu.......................................................   1     1   Fm3
.................................caucuccucucauugcagugcuU......................................................   1     1   Fm3
.................................caucuccucucauugcagugcuUu.....................................................   6     1   Fm3
.................................caucuccucucauugcagugcugua....................................................   2     0   Fm3
.................................caucuccucucauugcagugcuguaua..................................................   1     0   Fm3
..................................aucuccucucauugcagugc........................................................   4     0   Fm3
...............................................................gauaugcacuacauuguugaugaggu.....................   2     0   Fm3
.....................................................................cacuGcauuguugaugag.......................   10    1   Fm3
.....................................................................cacuacauuguugaugaggu.....................   4     0   Fm3
.....................................................................cacuacaGuguugaugaggu.....................   1     1   Fm3
.....................................................................cacuacauuguugaugagguggA..................   6     1   Fm3
.....................................................................cacuacauuguugaugaUguggc..................   1     1   Fm3
.....................................................................cacuacauuguugaugagguggc..................   66    0   Fm3
.....................................................................cacuacauuguugaugaAguggc..................   1     1   Fm3
......................................................................acuacauuguugaugaggu.....................   2     0   Fm3
......................................................................acuacauuguugaugagUug....................   1     1   Fm3
......................................................................acuacauuguugaugaggug....................   34    0   Fm3
......................................................................acuacauuguugaugagguA....................   2     1   Fm3
......................................................................acuacauuguugaugaUgugg...................   1     1   Fm3
......................................................................acuacauuguugaugagAugg...................   1     1   Fm3
......................................................................acuacauuguCgaugaggugg...................   1     1   Fm3
......................................................................acuaAauuguugaugaggugg...................   1     1   Fm3
......................................................................acuacauuguugaugagUugg...................   2     1   Fm3
......................................................................acuacauuguugaugaggugg...................   169   0   Fm3
......................................................................acuacauuguugaugaAgugg...................   1     1   Fm3
......................................................................acuacauuguugaugaUguggc..................   1     1   Fm3
......................................................................acuacauuguugaugagguggU..................   21    1   Fm3
......................................................................acuacauuguAgaugagguggc..................   3     1   Fm3
......................................................................acuacauuguugaugagguggA..................   23    1   Fm3
......................................................................acuacauuguugaugagAuggc..................   3     1   Fm3
......................................................................Ucuacauuguugaugagguggc..................   1     1   Fm3
......................................................................acuacauuguugaugaAguggc..................   4     1   Fm3
......................................................................acuacauuguugaugagguggc..................   771   0   Fm3
......................................................................acuacauuguugUugagguggc..................   1     1   Fm3
......................................................................aAuacauuguugaugagguggc..................   1     1   Fm3
......................................................................acuacauuguugaugCgguggc..................   1     1   Fm3
......................................................................acuacauuguugaugagCuggc..................   3     1   Fm3
......................................................................acuacauuguugaugaCguggc..................   5     1   Fm3
......................................................................acuacauuguugaugagUuggc..................   1     1   Fm3
......................................................................acuacauuguugaugagguggcU.................   87    1   Fm3

.................................caucuccucucauugcagugc........................................................   20    0   Fm2
.................................Uaucuccucucauugcagugc........................................................   2     1   Fm2
.................................caucuccucucauugcaCugc........................................................   1     1   Fm2
.....................................................................cacuacauuguugaugaggug....................   5     0   Fm2
.....................................................................cacuacauuguugaugaggugg...................   1     0   Fm2
.....................................................................cacuacauuguugaugagguggc..................   3     0   Fm2
......................................................................acuacauuguugaugaggug....................   5     0   Fm2
......................................................................acuacauuguugaugagUugg...................   1     1   Fm2
......................................................................acuacauuguugaugaggugg...................   7     0   Fm2
......................................................................acuacauuguugaugagguggc..................   155   0   Fm2
......................................................................acuacauuguAgaugagguggc..................   1     1   Fm2
......................................................................acuacauuguugaugaUguggc..................   2     1   Fm2
......................................................................acuacauuguugaugagCuggc..................   1     1   Fm2

.................................caucuccucucauugcagu..........................................................   1     0   Fm1
.................................caucuccucucauugcagugc........................................................   7     0   Fm1
```

```
                                 Star                                    Mature
agacaguggaaaugaagcagcagcuagcuuucucaucuccucucauugcagugcuguauaugugauaugcacuacauuguugaugagguggcaauguuggcuuuuaucau
............................................................ugugauaugcacuacauuguugaugaggug....................    2     0    Fm1
.................................................................uaugcacuacauuguugaugagC......................    1     1    Fm1
.....................................................................cacuacauuguugaugaggug....................    4     0    Fm1
.....................................................................cacuacauuguugaugUggugg...................    1     1    Fm1
.....................................................................cacuacauuguugaugaggugg...................    5     0    Fm1
.....................................................................cacuacauuguugaugagguggU..................    2     1    Fm1
.....................................................................cacuacauugCugaugagguggc..................    1     1    Fm1
.....................................................................cacuacauuguugaugagguggc..................    94    0    Fm1
.....................................................................cGcuacauuguugaugagguggc..................    1     1    Fm1
.....................................................................cacuacauuguugaugaggGggc..................    1     1    Fm1
.....................................................................cacuacauuguugaugaCguggc..................    1     1    Fm1
.....................................................................cacuacauuguugaugagguggA..................    4     1    Fm1
.....................................................................cacuacauuguugaugagguggcU.................    1     1    Fm1
......................................................................acuacauuguugaugaggugg...................    24    0    Fm1
......................................................................acuGcauuguugaugagguggc..................    6     1    Fm1
......................................................................acuacauuguugaugagguggU..................    8     1    Fm1
......................................................................acuacauuguugaugagAuggc..................    1     1    Fm1
......................................................................acuacauuguugaugagCuggc..................    2     1    Fm1
......................................................................Ucuacauuguugaugagguggc..................    1     1    Fm1
......................................................................acuacauuguugaugagguggc..................    141   0    Fm1
......................................................................acuacauuguugaugaggugCc..................    1     1    Fm1
......................................................................acuacauuguugaugagguggUa.................    1     1    Fm1
```
